# Use of a web-based educational intervention to improve knowledge of healthy diet and lifestyle in women with Gestational Diabetes Mellitus compared to standard clinic-based education

**DOI:** 10.1186/s12884-016-0996-7

**Published:** 2016-08-05

**Authors:** Padaphet Sayakhot, Mary Carolan-Olah, Cheryl Steele

**Affiliations:** 1 Victoria University, St Albans Campus, College of Health and Biomedicine, Building 4C, McKechnie Street, St Albans, Victoria 3021 Australia; 2 Diabetes Education Service, Endocrinology and Diabetes Centre, Western Health Sunshine Hospital, 176 Furlong Road, Sunshine, Victoria 3021 Australia

**Keywords:** Gestational diabetes mellitus, Knowledge, Understanding, Food choice, Management

## Abstract

**Background:**

This study introduced a web-based educational intervention for Australian women with gestational diabetes mellitus (GDM). The aim was to improve knowledge on healthy diet and lifestyle in GDM. Evaluation of the intervention explored women’s knowledge and understanding of GDM, healthy diet, healthy food, and healthy lifestyle, after using the web-based program compared to women receiving standard clinic-based GDM education.

**Methods:**

A total of 116 women, aged 18–45 years old, newly diagnosed with GDM, participated (Intervention (*n*) = 56 and control (*n*) = 60). Women were randomly allocated to the intervention or control groups and both groups attended a standard GDM education class. Group 1(Intervention) additionally used an online touch screen/computer program. All women completed a questionnaire following the computer program and/or the education class. All questions evaluating levels of knowledge had more than one correct answer and scores were graded from 0 to 1, with each correct component receiving a score, eg. 0.25 per each correct answer in a 4 answer question. Chi-square test was performed to compare the two groups regarding knowledge of GDM.

**Results:**

Findings indicated that the majority of women in the intervention group reported correct answers for “types of carbohydrate foods” for pregnant women with GDM, compared to the control group (62.5 % vs 58.3 %, respectively). Most women in both groups had an excellent understanding of “fruits and vegetables” (98.2 % vs 98.3 %), and the majority of women in the intervention group understood that they should exercise daily for 30 min, compared to the control group (92.9 % vs 91.7 %). Both groups had a good understanding across all categories, however, the majority of women in the intervention group scored all correct answers (score = 1) in term of foetal effects (17.9 % vs 13.3 %, respectively), maternal predictors (5.4 % vs 5 %), care requirements (39.3 % vs 23.3 %), GDM perceptions (48.2 % vs 46.7 %) and GDM treatment (67.9 % vs 61.7 %), compared to women in the control group.

**Conclusion:**

The study suggested that both approaches, standard education and standard education plus web-based program, resulted in excellent knowledge scores, but not statistically significant difference between groups. Multiple and immediate access to the web-based education program at home may prove useful as a source of reference for women with GDM. Future study comparing results pre and post intervention is needed.

**Trial registration:**

ACTRN12615000697583; Date registered: 03/07/2015; Retrospectively registered.

**Electronic supplementary material:**

The online version of this article (doi:10.1186/s12884-016-0996-7) contains supplementary material, which is available to authorized users.

## Background

Knowledge is considered as a significant component of health literacy [1]. Poor health literacy is associated with limited understanding of disease and may result in limited knowledge of disease management [2–7]. Recent studies have reported that many women misunderstand gestational diabetes mellitus (GDM), with women reporting that GDM only affects them during pregnancy, and that once the baby is born the complication is no longer a health threat [8, 9]. Although GDM usually resolves postpartum, there are long term consequences of obesity and risk of type 2 diabetes mellitus (DM) following diagnosis of GDM [10]. There is also strong evidence indicating that promoting healthy lifestyle habits such as weight loss, exercise, and healthy diet will reduce the risk of developing type 2 diabetes [11–13]. In addition, follow up doctor appointments, and postpartum Oral Glucose Tolerance Test (OGTT), are important for early diagnosis and prevention of type 2 DM in women with GDM [1].

Nowadays, the internet has become a very popular source of health information for pregnant women [14–18]. Many women utilise the internet as a primary resource for information about pregnancy, and as a means to help them deal with concerns, or to navigate pregnancy-related decisions, and to improve knowledge about pregnancy [16, 17, 19]. This popularity may relate to advantages, such as immediate access, usually at home, which avoids travel and is cost saving [20–23]. Pregnant women with GDM are one such group of users. The present study introduced a web-based education program for women with GDM which aimed to support GDM education and self-management. The program was developed in close cooperation with potential users [24] and is based on national guidelines for diabetes care.

Finally, although a number of studies have reported on knowledge evaluation among women with type 1 and type 2 DM, literature related to knowledge evaluation among women with GDM is limited, and no studies were located which used a web-based educational intervention to improve the knowledge about GDM. For these reasons, this study focused on evaluating knowledge of GDM, self-management, healthy diet, exercise, and lifestyle habits.

The aim of this study was to explore whether or not the knowledge/understanding of GDM could be improved by introducing this additional web-based education compared to standard GDM education alone.

## Methods

### Study design

This study was a randomised controlled trial (RCT) study comparing two groups of participants. All protocols were approved by the WH HREC. Informed written consent was obtained from each participant prior to enrolment. This study was carried out at the Maternity Diabetes Clinic of Western Health Sunshine Hospital from December 2014-May 2015.

### Study population

#### Inclusion and exclusion criteria

Pregnant women aged between 18–45 years old, newly diagnosed with GDM, with a singleton pregnancy, who attended the maternity diabetes clinic at Western Health Sunshine Hospital, were invited to participate in the study. Pregnant women, who had pre-existing diabetes (types 1 and 2), or were unable to write and understand English, were excluded from the study. Participants in both groups (Intervention Group and Control Group) were expected to meet the same criteria.

### Study setting

Women were randomly allocated to either an intervention group or a control group:

#### Intervention group

Women in the intervention group attended the standard clinic-based education and additionally used the web-based education intervention. Access to the web-based education intervention (A touch screen computer) took place at the Maternity diabetes clinic at Western Health Sunshine Hospital, supervised by the researcher. The program provided information on healthy diet and healthy lifestyle and included pictures and simple instructions. The program took 15–30 min to complete. Women were also given the URL link for the website, so they could access the website as they wished at home.

#### Control group

Women in the control group attended the standard clinic-based education class alone. The single, group education class, lasting 1.5 h, is run by Western Health’s dieticians and diabetes educator nurses. Generally 5–8 newly diagnosed pregnant women with GDM attend. Session and content is based on healthy diet, exercise, healthy lifestyle, and blood glucose level monitoring.

### Study tools and measurement

A structured questionnaire and a web-based educational intervention/touch screen computer were used in this study.

#### Questionnaire

**Fig. 1:**
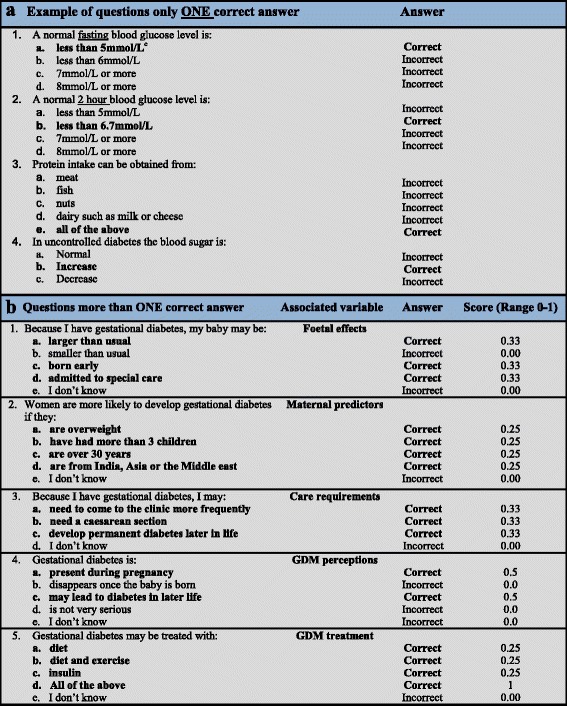
Sample questions/statements about diabetes with one and more than one correct answer. ^c^All statement in “*bold*” are represented a correct answer

The questionnaire included health questionnaires developed/adapted by the authors and used in previous studies of Australian women [25], which included demographic information, health, knowledge of GDM, knowledge of testing blood glucose level, knowledge of food choice and self-managing GDM. The Diabetes Knowledge Scale [26] was specifically adapted to focus on pregnancy and GDM, and used in a previous study [25] to assess knowledge about GDM. Most questions had one correct answer and were scored as correct/incorrect. (eg, “in uncontrolled diabetes the blood sugar is: (a) Normal; (b). Increased; and (c). Decreased”. The correct answer is *b. increased*) (Fig. 1a). Questions evaluating levels of knowledge had more than one correct answer (eg, “Because I have gestational diabetes, my baby may be: (a). Larger than usual; (b). Smaller than usual; (c). Born early; (d). Admitted to special care; (e). I don’t know”. The correct answer is *a, c* and *d*) (Fig. 1b). A score was given for each correct answer to a total of 1 per question (Score 0.33 = one correct out of 3 answers; Score 0.66 = two correct out of 3 answers; Score 0.25 = one correct out of 4 answers; Score 0.5 = two correct out of 4 answers; Score 0.75 = three correct out of 4 answers; and Score 1 = all correct answers). Detailed information about the questionnaire and scoring system is included in Fig. 1a and b.

#### Website design/intervention program

The web-based education intervention contained four modules about GDM and four information resources. All information on the website was decided by a research panel comprising of dietician, endocrinologist, diabetes nurse educator, midwife and researchers. The website was developed and tested prior to this study and was described in our previous research [24]. The four modules covered: (1) healthy food choices, (2) healthy habits/healthy lifestyle, (3) emotions, family and food, and (4) testing blood glucose level. The four information resources were: (1) What is gestational diabetes?, (2) Healthy eating and exercise in GDM, (3) What to do if you’re still hungry?, and (4) A guide to healthy shopping.

### Recruitment

Women, who met the study criteria, were recruited from the Maternity Diabetes Clinic. Once women agreed to participate, they were randomly allocated to the intervention group, or control group by using computer-generated random numbers [27] (Additional file 1). Written consent was obtained prior to participation. Women in the intervention group were invited to complete a questionnaire after they had finished the standard education class plus web-based education. Meanwhile, the control group were invited to complete a questionnaire once they had finished the standard education class at the hospital. All women completed a questionnaire at the hospital (Details of recruitment procedure were shown in Additional file 2).

### Sample size

Power analysis was performed for sample size estimation. To ensure a power of 80 % and a significant level of 0.05 for each group, the study required a minimum of 50 participants per group and a maximum of 70 per group. In order to detect minimum differences in all aims, we aimed to recruit a minimum of 50 participants per groups, resulted in 56 women in the intervention group and 60 women for the control group.

### Data analysis

Analysis was performed using SPSS software (version 20.0; SPSS). Crosstabs, frequencies and descriptive statistics were used to summarize demographics. Authors also consulted with an expert biostatistician. Crosstabs with Chi-square test and Exact Chi-square were used to analyse data. *P* ≤ 0.05 was considered statistically significant. Results were reported as frequencies and percentage.

## Results

### Participant rate

The present study sample includes 116 women, with 56 (48 %) of the participants in the intervention group and 60 (52 %) allocated to the control group. The researcher approached a total of 130 pregnant women with GDM, who expressed interest in participation. After screening for eligibility, there were 116 women, who were eligible for the study, 5 (3.8 %) women had pre-existing diabetes mellitus, 2(1.5 %) women could not speak and write English, 2 (1.5 %) women did not complete the questionnaire, and 5(3.8 %) requested to complete the questionnaire at home, these women were excluded from the study. Therefore, the response rate for this study was 89.2 %.

### Demographic characteristics

The majority of participants in the intervention group were aged between 31–35 years old, while control group participants were aged 26–30 years (50 % and 40 %, respectively). More than one third (36.7 %) of participants in the control group were born in Australia and New Zealand, whereas, one third (33.3 %) of the women in the intervention group were born in India, Sri Lanka and Bangladesh. The majority of women who migrated to Australia had lived in Australia less than/equal to 10 years (82.9 % and 64.1 %, respectively). One third of the participants in both intervention and control groups were Christian (32.1 % and 33.3 %, respectively). Educational completion revealed that nearly one third of women had completed a bachelor degree (33.9 % and 21.7 %, respectively), one third (30.4 % and 36.7 %) of women worked full time, and one third were home duties (28.6 % and 33.3 %). The majority of the women in both groups (82.1 % and 71.7 %, respectively) were married (Table 1).

**Table 1:** Demographic characteristics

	Groups	
	Intervention *n* = 56 (%)	Control *n* = 60 (%)
Age:		
ᅟ- ≤25 years	5 (8.9)	5 (8.3)
ᅟ- 26-30 years	16 (28.6)	24 (40)
ᅟ- 31-35 years	28 (50)	18 (30)
ᅟ- ≥36 years	7 (12.5)	13 (21.7)
Country of birth:		
ᅟ- Australia, New Zealand	12 (22.2)	22 (36.7)
ᅟ- India, Sri Lanka, Bangladesh	18 (33.3)	10 (16.7)
ᅟ- China, Hong Kong, Taiwan, Nepal	6 (11.1)	3 (5.0)
ᅟ- Vietnam, Laos	3 (5.6)	11(18.3)
ᅟ- Ethiopia, Eritrea, Sudan, Congo, Somalia	6 (11.1)	4 (6.7)
ᅟ- Philippine, Malaysia, Singapore	2 (3.7)	3 (5.0)
ᅟ- Other	7 (13.0)	7 (11.6)
Been in Australia:		
ᅟ- ≤10 years	34 (82.9)	25 (64.1)
ᅟ- 11 – 20 years	2 (4.8)	6 (15.3)
ᅟ- ≥21 years	5 (12.1)	8 (20.5)
Religion:		
ᅟ- Christian	18 (32.1)	20 (33.3)
ᅟ- Hindu	8 (14.3)	7 (11.7)
ᅟ- Muslim/Islam	9 (16.1)	5 (8.3)
ᅟ- No religion	13 (23.2)	12 (20.0)
ᅟ- Other: Sikh, Catholic, Buddhist	8 (14.3)	16 (26.7)
Education:		
ᅟ- Year 11 or below	3 (5.3)	7 (11.6)
ᅟ- Year 12 or equivalent	10 (17.9)	11 (18.3)
ᅟ- Certificate, advanced diploma or diploma	13 (23.2)	16 (26.7)
ᅟ- Bachelor degree	19 (34.0)	13 (21.7)
ᅟ- Postgraduate degree (e.g. Graduate diploma, Masters or PhD)	11 (19.6)	13 (21.7)
Work status:		
ᅟ- Full time	17 (30.4)	22 (36.7)
ᅟ- Part time	12 (21.4)	8 (13.2)
ᅟ- No paid work	18 (32.1)	14 (23.3)
ᅟ- Other	9 (16.1)	16 (26.7)
Occupation:		
ᅟ- Professional, high level manager or administrator	10 (17.9)	5 (8.3)
ᅟ- Manager or equivalent	4 (7.1)	8 (13.3)
ᅟ- Clerical, service worker or sales	9 (16.1)	19 (31.7)
ᅟ- Student (full time)	4 (7.1)	1 (1.7)
ᅟ- Home duties	16 (28.6)	20 (33.3)
ᅟ- No paid work	7 (12.5)	4 (6.7)
ᅟ- Other	6 (10.7)	3 (5.0)
Income:		
ᅟ- No income	5 (8.9)	6 (10.0)
ᅟ- < $60,000	16 (28.6)	19 (31.7)
ᅟ- $60,000-$80,000	10 (17.9)	16 (26.7)
ᅟ- Don’t know	13 (23.2)	4 (6.6)
ᅟ- I don’t want to answer	12 (21.4)	15 (25.0)
Marital status:		
ᅟ- Married	46 (82.1)	43 (71.7)
ᅟ- Defacto	5 (8.9)	12 (20)
ᅟ- Other	5 (8.9)	5 (8.3)

### Knowledge of gestational diabetes mellitus (GDM)

**Fig. 2:**
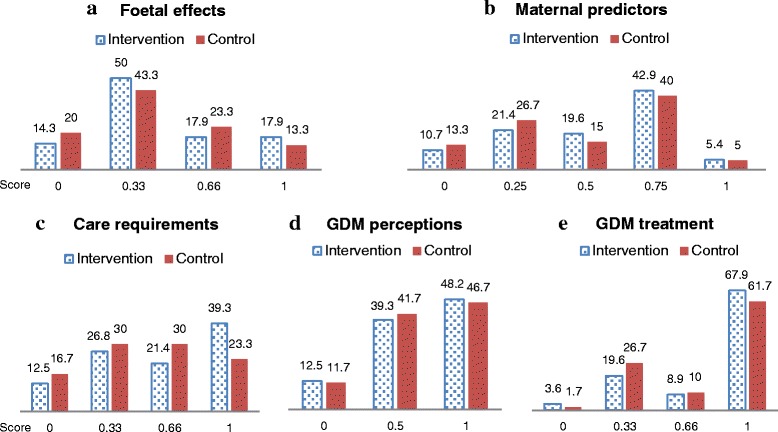
Knowledge of gestational diabetes. Score are range from 0–1; Score 0 = incorrect answer or don’t know the answer; Score 0.33 = one correct answer out of 3 answers; Score 0.66 = two correct answers out of 3 answers; Score 0.25 = one correct answer out of 4 answers; Score 0.5 = one correct answer out of 2 answers; Score 0.75 = three correct answers out of 4 answers; and Score 1 = all correct answers

General knowledge about GDM includes: Knowledge on foetal effects, maternal predictors, care requirements during pregnancy, GDM perceptions and GDM treatment. The correct answer for questions ranged from score 0 to 1 (see Fig. 2 for detail). Results were reported as: (1). “Poor” if the score was <0.25; (2). “Limited” if the score ranged from 0.25 to <0.5; (3). “Good” if the score ranged from 0.5 to 0.75; and (4). “Excellent” if the score was = 1. Findings indicated that nearly half of women in both groups had a limited understanding of GDM affects on the baby scoring 0.33 (50 % and 43.3 %, respectively). Few women had an excellent understanding of how GDM affected the baby with 17.9 % of women in the intervention group achieving a full score (score =1), compared to just 13.3 % of women in the control group (Fig. 2a).

Both groups had a good understanding of maternal predictors for GDM, with the majority of women in the intervention group scoring = 0.75 compared to the control group (42.9 % and 40 %, respectively) (Fig. 2b). The majority of participants in the intervention group had an excellent understanding of care requirements during pregnancy (39.3 % and 23.3 %, respectively), GDM perceptions (48.2 % and 46.7 %), and GDM treatment (67.9 % and 61.7 %) compared to the control group (Fig. 2c, d and e).

In addition, the overall findings for all categories indicated that more women in the intervention group had achieved a full score (score =1), compared to women in the control group.

### Knowledge of food choice and exercise during pregnancy

Food choices and exercise for women with GDM during pregnancy were understood similarly in both groups. The majority of women in the intervention group reported correct answers for “types of carbohydrate foods” for pregnant women with GDM, compared to the control group (62.5 % and 58.3 %, respectively). Most participants in both groups had an excellent understanding of fruits and vegetables (98.2 % and 98.3 %), and the majority of participants in both groups understood how exercise in GDM helps to control blood glucose and improve baby’s health (98.2 % and 98.3 %). Most women in both groups correctly identified “walking, swimming and yoga” as recommended exercises during pregnancy (96.4 % and 96.7 %) and the majority of women in the intervention group understood that they should exercise daily for 30 min, compared to the control group (92.9 % and 91.7 %). Differences were not reported between the two groups for all categories (*p* > 0.05) (Table 2). In addition, only one third of women in both groups reported correct answers in respect to moderate exercise (37.5 % and 40 %), while the majority of women in both groups misunderstood the amount of exercise required (50 % and 58.3 %) and a small number did not know the answer regarding exercise during pregnancy (12.5 % and 1.7 %).

**Table 2:** Knowledge of Food choice and exercise for pregnant women with gestational diabetes mellitus

	Groups		*p* value^a^
	Intervention *n* (%)	Control *n* (%)	
The preferred type of carbohydrate food			
ᅟ- Correct	35 (62.5)	35 (58.3)	0.647
ᅟ- Incorrect	21 (37.5)	25 (41.7)	
Form of fruits and vegetables are better			
ᅟ- Correct	55 (98.2)	59 (98.3)	0.961
ᅟ- Incorrect	1 (1.8)	1 (1.7)	
Protein intake obtained			
ᅟ- Correct	39 (69.6)	47 (78.3)	0.285
ᅟ- Incorrect	17 (30.4)	13 (21.7)	
Best type of protein			
ᅟ- Correct	38 (67.9)	47 (78.3)	0.203
ᅟ- Incorrect	18 (32.1)	13(21.7)	
A balanced diet should have			
ᅟ- Correct	40 (71.4)	44 (73.3)	0.819
ᅟ- Incorrect	16 (28.6)	16 (26.7)	
Exercise in GDM helps to control blood glucose and improve baby’s health			
ᅟ- Correct	55 (98.2)	59 (98.3)	0.961
ᅟ- Incorrect	1 (1.8)	1 (1.7)	
Exercises that are recommended during pregnancy			
ᅟ- Correct	54 (96.4)	58 (96.7)	0.944
ᅟ- Incorrect	2 (3.6)	2 (3.3)	
How hard can you exercise during pregnancy?			
ᅟ- Correct	20 (35.7)	24 (40)	0.635
ᅟ- Incorrect	36 (64.3)	36 (60)	
How long should you exercise per day?			
ᅟ- Correct	43 (76.8)	48 (80)	0.674
ᅟ- Incorrect	13 (23.2)	12 (20)	
Should I exercise if I am overweight and unfit			
ᅟ- Correct	48 (85.7)	56 (93.3)	0.178
ᅟ- Incorrect	8 (14.3)	4 (6.7)	
How can I increase my daily exercise?			
ᅟ- Correct	42 (75)	50 (83.3)	0.268
ᅟ- Incorrect	14 (25)	10 (16.7)	
You should exercise daily for 30 min			
ᅟ- Correct	52 (92.9)	55 (91.7)	0.811
ᅟ- Incorrect	4 (7.1)	5 (8.3)	

^a^Chi-square test

### Knowledge of GDM management

Normal fasting blood glucose levels were well understood by both groups (91.1 % and 91.7 % respectively). A normal 2 h post-prandial (after meals) blood glucose level was also reported correctly in both groups (91.1 % and 90 %). The majority of women in both groups knew what they needed to do if their blood glucose level was high on one occasion (80.4 % and 80 %) and if it was high in two occasions in one week (60.7 % and 60 %). Both groups had a good understanding of testing blood glucose level if they were sick (94.6 % and 95 %) (Table 3). Significant differences were reported in “time of testing blood glucose level” *p* = 0.026 as more women in the control group (86.7 %) reported correct answers compared to women in the intervention group (69.6 %). Statistically significant differences were also reported in “pricking their fingers for blood glucose test” *p* = 0.016, as the majority of women in the control group reported more correct answers than women in the intervention group (75 % vs 53.6 %, respectively).

**Table 3:** Knowledge of testing blood glucose level

	Groups		*p* value^a^
	Intervention *n* = 56 (%)	Control *n* = 60 (%)	
A normal fasting (on an empty stomach) blood glucose level is:			
ᅟ- Correct	51 (91.1)	55 (91.7)	0.909
ᅟ- Incorrect	5 (8.9)	5 (8.3)	
A normal 2 h blood glucose level is:			
ᅟ- Correct	51 (91.1)	54 (90)	0.844
ᅟ- Incorrect	5 (8.9)	6 (10)	
I should test my blood glucose level (eg, in the morning before breakfast/2 h after meals):			
ᅟ- Correct	39 (69.6)	52 (86.7)	0.026^b^
ᅟ- Incorrect	17 (30.4)	8 (13.3)	
What do I do if my blood glucose level is high on one occasion?			
ᅟ- Correct	45 (80.4)	48 (80)	0.962
ᅟ- Incorrect	11 (19.6)	12 (20)	
What do I do if my blood glucose level is high on two occasions in one week?			
ᅟ- Correct	34 (60.7)	36 (60)	0.937
ᅟ- Incorrect	22 (39.3)	24 (40)	
Should I take my blood glucose level if I am feeling sick and haven’t eaten?			
ᅟ- Correct	53 (94.6)	57 (95)	0.931
ᅟ- Incorrect	3 (5.4)	3 (5)	
When you prick your finger, you should:			
ᅟ- Correct	30 (53.6)	45 (75)	0.016^b^
ᅟ- Incorrect	26 (46.4)	15 (25)	

^a^Chi-square test

^b^ *p* ≤ 0.05 was considered statistically significant

Checking blood glucose levels regularly was well understood by both groups (96.4 % and 96.7 %, respectively). The majority of women in the intervention group understood the influence of controlling their blood glucose levels on the baby, compared to the control group (87.5 % and 86.7 %). Statistically significant differences were reported in “controlling GDM” by changing to a healthy diet and exercise (*p* = 0.036); and significant difference in managing when hungry in between meals *p* = 0.008 (Table 4).

**Table 4:** Management of gestational diabetes mellitus

	Groups		*p* value^a^
	Intervention *n* (%)	Control *n* (%)	
You should check your blood glucose levels			
ᅟ- Correct	54 (96.4)	58 (96.7)	0.944
ᅟ- Incorrect	2 (3.6)	2 (3.3)	
Controlling your blood glucose levels:			
ᅟ- Correct	49 (87.5)	52(86.7)	0.894
ᅟ- Incorrect	7 (12.5)	8(13.3)	
If there is a social occasion, such as a party, you should:			
ᅟ- Correct	40 (71.4)	51 (85)	0.076
ᅟ- Incorrect	16 (28.6)	9 (15)	
When your blood glucose levels are high:			
ᅟ- Correct	47 (83.9)	51 (85)	0.873
ᅟ- Incorrect	9 (16.1)	9 (15)	
To control blood glucose effectively you should			
ᅟ- Correct	35 (62.5)	44 (73.3)	0.211
ᅟ- Incorrect	21 (37.5)	16 (26.7)	
GDM can be controlled by:			
ᅟ- Correct	48 (85.7)	58 (96.7)	0.036^b^
ᅟ- Incorrect	8 (14.3)	2 (3.3)	
When you are hungry in between meals:			
ᅟ- Correct	32 (57.1)	48 (80)	0.008^b^
ᅟ- Incorrect	24 (42.9)	12 (20)	

^a^Chi-square test and Chi-square exact test

^b^ *p* ≤ 0.05 was considered statistically significant

In addition, the majority (76.8 %) of women in the intervention group understood that they should get a follow up glucose test at 6 weeks set up compared to control group (81.7 %). One third (28.3 %) of participants in the control group misunderstood complications of GDM following birth, compared to nearly half (48.2 %) of participants in the intervention group. There was no statistically significant difference between the two groups.

### Association of education levels and knowledge about GDM

The majority of women in both groups with tertiary education had the highest knowledge scores (score = 1) in term of knowledge on care requirements during pregnancy (27.6 % vs 3.4 %), GDM perceptions (36.2 % vs 11.2 %) and GDM treatment (49.1 % vs 15.5 %), compared to the women with primary or secondary education. There was no statistically significant difference between the education levels (*p* > 0.05).

In addition, more women with tertiary education reported correct answers for “fruits and vegetables” (72.4 % vs 24.1 %, *p* = 0.026) and “how long they should exercise per day” (60.3 % vs 18.1 %, *p* = 0.090), compared to women with primary or secondary education. The majority of women who had tertiary education, had an excellent understanding of “the preferred type of carbohydrate food” (47.4 % vs 12.9 %, *p* > 0.05), and “how hard they can exercise during pregnancy” (27.6 % vs 10.3 %, *p* > 0.05), compared to women with lower education level. More women with tertiary education reported correct answers for “time to test blood glucose level” (62.1 % vs 16.4 %, *p* = 0.007) and “pricking fingers for blood glucose test” (42.2 % vs 22.4 %, *p* = 0.009), compared to women with lower education level.

## Discussion

There are thousands of websites on the Internet that provide nutrition information, including websites that mention the GDM diet. Most of these websites provide static content and are difficult to understand. The present study introduced a web-based education that provides GDM educational information with pictures and simple structures, and convenient ways for self-learning. This study estimated knowledge levels of women who used the web-based education intervention in addition to the standard education class, compared to women who attended the standard education class alone. It also sought to understand if the web-based education was a useful support and additional source of information for women newly diagnosed with GDM. The overall findings of this study indicated that both groups of participants had a good understanding of GDM, food choices, exercise during pregnancy and GDM management. The most probable reason for this finding is that all participants in the intervention group completed the questionnaire following the use of the website and attended the GDM education class before answering questionnaires, and all participants in the control group also completed the questionnaire after they attended the GDM class. Therefore, the information that the women had received from the internet and the class may improve their knowledge about GDM. Future research involving a comparison between pre and post intervention knowledge for both the website and education class would help to clarify this point.

Although both groups of participants had a good understanding across all categories, the majority of women in the intervention group reported excellent understanding of GDM in term of foetal effects, maternal predictions, care requirements, GDM predictions and GDM treatment compared to women in the control group (score =1) (see Fig. 2). This finding suggests that our current online education program does not make an immediate impact on women’s knowledge by improving their understanding of GDM. However, the program has potential as a support for the current education approach, and may need some revision to fill this potential. The literature supports this potential as Moore et al. [28], who studied an internet-based nutrition education program and explored its effect on weight, blood pressure, and eating habits after 12 months of participation. They found the effectiveness of online programs for health, delivered via the Internet, can provide benefits to large numbers of subjects at low cost and may help address the nutritional public health crisis [28]. Similarly, the present web-based education intervention can be used as a source of information for women with GDM.

In addition, another objective of this study was to estimate how much women understand healthy diet/ healthy food and healthy lifestyle following the use of a web-based education compared to women with a standard clinic-based education. The findings indicated that both groups of participants had an excellent understanding of healthy diet and food choices, except one question regarding types of carbohydrate food. This question was answered correctly by the majority of women in the intervention, compared to the control group. The possible reason for this finding is that the information on the online educational website may be easier to understand compared to the education class. As our online education uses pictures of food choices, healthy diet, appropriate portions, healthy lifestyle and exercise, and also simple instructions describing what are healthy diets, food portions, and healthy lifestyle. The present findings are supported by number of studies using web-based nutrition education programs for weight management which confirm that e-learning education/web-based education improves knowledge and promotes behaviours associated with weight loss [28–32].

Other key findings indicated that the poorest knowledge levels were observed for a question on “how hard can they exercise during pregnancy?” with most women in both groups reporting incorrect answers. Most women understood that they should only do gentle exercise during pregnancy. Although the web-based education and class education provided all the necessary information to answer the questions, many women did not assimilate this information. The possible reason for this finding may be the level of education that women in this study had completed, as the majority of women in both groups had lower than average education levels. Women with primary education had the lowest knowledge scores, whereas, women who had bachelor degree/post graduate degree level education showed highest knowledge scores (score =1). Poor knowledge seemed to be associated with lower education level. This finding is supported by a recent study on evaluation of knowledge of GDM among Malaysian women, which found that educational level was a strong influencing factor for level of knowledge across all knowledge domains. This study similarly found poorest knowledge of GDM among women with lower education levels and highest scores in women with higher educational levels [33].

Moreover, women with lower education level might experience barriers in understanding the web-based education and may have difficulty communicating with diabetes educators and dieticians. The findings from this study confirmed previous research outcomes that showed the education had a strong impact on health literacy. Low educational level leads to low health literacy which results in limited knowledge about conditions [25, 34–36]. Participants with low health literacy may have difficulty understanding patient-orientated health literature, medication instructions, appointment cards from clinic and hospital signage [37].

Finally, the present study found that one question regarding “where to prick the fingers for testing blood glucose level?” - was answered incorrectly by nearly half of women in the intervention group compared to less than one third of women in the control group. A statistically significant difference was reported on questions regarding pricking fingers for testing blood glucose (*p* < 0.05). The probable reason for this finding is that women were new to the website and may have misunderstood the question. However, although this study collected questionnaire information at one time only, women can access the website at home at any time, whereas, women in the control group attend the education class once only. These women may find a difficulty following a self-management plan after they attend the class, and this may increase the incidence of complications, and decrease coping skills. Similar concerns were raised in a study in Malaysian women where GDM participants reported poor knowledge and difficulties related to the management of GDM [33].

### Implications

Practice implication:

There is a need to translate the findings of this study into practice as our findings reveal a number of areas that require greater attention. In particular, the online education program/web-based needs to be improved, as follows:

1. Providing clearer information on the amount of exercise during pregnancy,
2. Providing clearer information on pricking fingers for blood glucose test, and
3. Providing clearer information on the correct time for testing blood glucose levels.

Additionally, women with GDM should be able to attend the hospital education class more than once as some women displayed incorrect knowledge after a single class.

These improvements have the capacity to improve outcomes as better knowledge about GDM is associated with better management of GDM.

### Limitations

Although, the desirable aims of the study have been achieved, there are some limitations related to the study. This study was conducted in single centre so results of this study cannot be generalized.

The web-based education was evaluated in a randomized controlled trial comparing two groups of the women: (1) intervention group (women attended the clinic-based education class and additionally used the web-based education intervention) and (2) control group (women attended the clinic-based education class only). There was a limitation in the method of this study due to baseline information/pre-intervention was not collected. Future study comparing results pre- and post-intervention, or a study comparing standard education with web-based education is suggested.

The number of participants in the intervention group was smaller than the number in the control group. This was because it was difficult to recruit participants for the intervention group as the women have to complete the questionnaire after they have used the program, which was inconvenient for them to do at the maternity clinic. Some women partially completed the questionnaire or requested to complete the questionnaire at home and their data was not considered. Therefore, an online survey or reply-paid envelop is recommended for future studies.

## Conclusions

Both groups of women had excellent results, and results were not significantly improved by the intervention, nonetheless the online educational program has potential to improve GDM education and may provide convenient ways for self-learning about GDM for women. Revision of the program to tailor it to the women’s needs in terms of reinforcing information and key messages might be the way forward. Future study comparing results pre and post intervention is needed.

## Abbreviations

DM, diabetes mellitus; GDM, gestational diabetes mellitus; WH HREC, Western health human research committees

## Additional files

Additional file 1: Results of Random Numbers by using a Random Number Generator. (XLSX 9 kb) Additional file 2: Flow chart of recruitment procedure. (PDF 103 kb)

## References

[1] Baker DW (2006). The meaning and the measure of health literacy. J Gen Intern Med.

[2] Williams MV, Baker DW, Parker RM, Nurss JR (1998). Relationship of functional health literacy to patients' knowledge of their chronic disease. A study of patients with hypertension and diabetes. Arch Intern Med.

[3] Ostlund I, Hanson U, Bjorklund A, Hjertberg R, Eva N, Nordlander E (2003). Maternal and fetal outcomes if gestational impaired glucose tolerance is not treated. Diabetes Care.

[4] Langer O, Yogev Y, Xenakis EM, Rosenn B (2005). Insulin and glyburide therapy: dosage, severity level of gestational diabetes, and pregnancy outcome. Am J Obstet Gynecol.

[5] Guralnik JM, Land KC, Blazer D, Fillenbaum GG, Branch LG (1993). Educational status and active life expectancy among older blacks and whites. N Engl J Med.

[6] Pawlak R (2005). Economic considerations of health literacy. Nurs Econ.

[7] Dewalt DA, Berkman ND, Sheridan S, Lohr KN, Pignone MP (2004). Literacy and health outcomes: a systematic review of the literature. J Gen Intern Med.

[8] Kapustin JF (2008). Postpartum management for gestational diabetes mellitus: policy and practice implications. J Am Acad Nurse Pract.

[9] Kim C, McEwen LN, Piette JD, Goewey J, Ferrara A, Walker EA (2007). Risk perception for diabetes among women with histories of gestational diabetes mellitus. Diabetes care.

[10] Reece EA (2010). The fetal and maternal consequences of gestational diabetes mellitus. J Matern Fetal Neonatal Med.

[11] England LJ, Dietz PM, Njoroge T, Callaghan WM, Bruce C, Buus RM et al. Preventing type 2 diabetes: public health implications for women with a history of gestational diabetes mellitus. Am J Obstet Gynecology. 2009;200(4):365.e1-8. doi:10.1016/j.ajog.2008.06.031. 10.1016/j.ajog.2008.06.031 18691691

[12] Knowler WC, Barrett-Connor E, Fowler SE, Hamman RF, Lachin JM, Walker EA (2002). Reduction in the incidence of type 2 diabetes with lifestyle intervention or metformin. N Engl J Med.

[13] Tuomilehto J, Lindstrom J, Eriksson JG, Valle TT, Hamalainen H, Ilanne-Parikka P (2001). Prevention of type 2 diabetes mellitus by changes in lifestyle among subjects with impaired glucose tolerance. N Engl J Med.

[14] Gao LL, Larsson M, Luo SY (2013). Internet use by Chinese women seeking pregnancy-related information. Midwifery.

[15] Lagan BM, Sinclair M, Kernohan WG (2010). Internet use in pregnancy informs women's decision making: a web-based survey. Birth.

[16] Romano AM (2007). A Changing Landscape: Implications of Pregnant Women’s Internet Use for Childbirth Educators. J Perinat Educ.

[17] Song FW, West JE, Lundy L, Smith-Dahmen N (2012). Women, Pregnancy, and Health Information Online: The Making of Informed Patients and Ideal Mothers. Gend Soc.

[18] Eysenbach G, Powell J, Kuss O, Sa ER (2002). Empirical studies assessing the quality of health information for consumers on the world wide web: a systematic review. Jama.

[19] Lagan BM, Sinclair M, Kernohan WG (2011). What is the impact of the Internet on decision-making in pregnancy? A global study. Birth.

[20] Bell JA, Patel B, Malasanos T (2006). Knowledge improvement with web-based diabetes education program: brainfood. Diabetes Technol Ther.

[21] Noh JH, Cho YJ, Nam HW, Kim JH, Kim DJ, Yoo HS (2010). Web-based comprehensive information system for self-management of diabetes mellitus. Diabetes Technol Ther.

[22] Meesters JJ, de Boer IG, van den Berg MH, Fiocco M, Vliet Vlieland TP (2012). Evaluation of a website providing information on regional health care services for patients with rheumatoid arthritis: an observational study. Clin Rheumatol.

[23] Heinrich E, de Nooijer J, Schaper NC, Schoonus-Spit MH, Janssen MA, de Vries NK (2012). Evaluation of the web-based Diabetes Interactive Education Programme (DIEP) for patients with type 2 diabetes. Patient Educ Couns.

[24] Carolan-Olah M, Steele C, Krenzin G (2015). Development and initial testing of a GDM information website for multi-ethnic women with GDM. BMC Pregnancy Childbirth.

[25] Carolan M, Steele C, Margetts H (2010). Knowledge of gestational diabetes among a multi-ethnic cohort in Australia. Midwifery.

[26] Dunn SM, Bryson JM, Hoskins PL, Alford JB, Handelsman DJ, Turtle JR (1984). Development of the diabetes knowledge (DKN) scales: forms DKNA, DKNB, and DKNC. Diabetes Care.

[27] Urbaniak GC, Plous S. Research Randomizer Version 4.0. Computer software. 2014. http://www.randomizer.org/. Accessed 8 Dec 2014.

[28] Moore TJ, Alsabeeh N, Apovian CM, Murphy MC, Coffman GA, Cullum-Dugan D (2008). Weight, blood pressure, and dietary benefits after 12 months of a Web-based Nutrition Education Program (DASH for health): longitudinal observational study. J Med Internet Res.

[29] Heetebry I, Hatcher M, Tabriziani H (2005). Web based Health Education, E-learning, for weight management. J Med Syst.

[30] Safran Naimark J, Madar Z, Shahar DR (2015). The impact of a Web-based app (eBalance) in promoting healthy lifestyles: randomized controlled trial. J Med Internet Res.

[31] Ajie WN, Chapman-Novakofski KM (2014). Impact of computer-mediated, obesity-related nutrition education interventions for adolescents: a systematic review. J Adolesc Health.

[32] Neve M, Morgan PJ, Jones PR, Collins CE (2010). Effectiveness of web-based interventions in achieving weight loss and weight loss maintenance in overweight and obese adults: a systematic review with meta-analysis. Obes Rev.

[33] Hussain Z, Yusoff ZM, Sulaiman SA (2015). Evaluation of knowledge regarding gestational diabetes mellitus and its association with glycaemic level: A Malaysian study. Prim Care Diabetes.

[34] Gazmararian JA, Williams MV, Peel J, Baker DW (2003). Health literacy and knowledge of chronic disease. Patient Educ Couns.

[35] Kalichman SC, Benotsch E, Suarez T, Catz S, Miller J, Rompa D (2000). Health literacy and health-related knowledge among persons living with HIV/AIDS. Am J Prev Med.

[36] Powell CK, Hill EG, Clancy DE (2007). The relationship between health literacy and diabetes knowledge and readiness to take health actions. Diabetes Educ.

[37] Dani KA, Stobo DB, Capell HA, Madhok R (2007). Audit of literacy of medical patients in north Glasgow. Scott Med J.

