# Supplementary figures and images for: Use of a web-based educational intervention to improve knowledge of healthy diet and lifestyle in women with Gestational Diabetes Mellitus compared to standard clinic-based education
Source: BMC Pregnancy Childbirth. 2016 Aug 5;16:208. doi: 10.1186/s12884-016-0996-7 (PMC4974775; doi:10.1186/s12884-016-0996-7)

**Additional file 2** Flow chart of recruitment procedure.

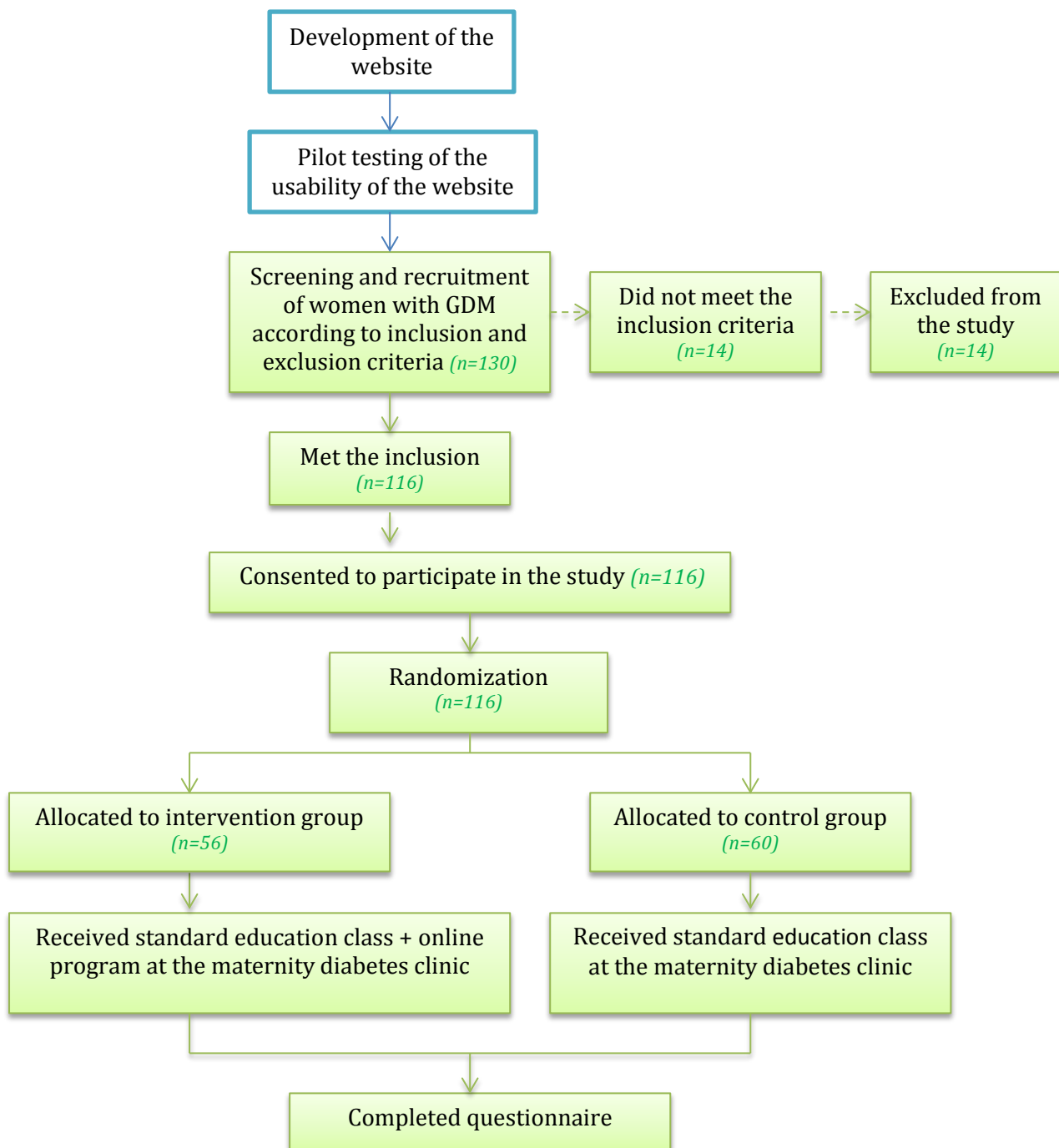

Supplement: Additional file 2: — Flow chart of recruitment procedure. (PDF 103 kb) [file 12884_2016_996_MOESM2_ESM.pdf]
